# A Novel Method of Natural Orifice Specimen Extraction Surgery (NOSES) during Laparoscopic Anterior Resection for Rectal Cancer

**DOI:** 10.1155/2021/6610737

**Published:** 2021-01-25

**Authors:** Jun Wang, Jun Hong, Qianwei Wang, Fen Luo, Fenghua Guo

**Affiliations:** Department of General Surgery, Huashan Hospital, Fudan University, Shanghai 200090, China

## Abstract

We propose a modification to the reconstruction method of natural orifice specimen extraction surgery (NOSES) during laparoscopic anterior resection (LAR) for rectal cancer (RC) and evaluated its feasibility and short-term safety by comparing surgical and postoperative outcomes with those of conventional LAR. Twenty patients with RC underwent “double-purse” NOSES-LAR from October 2017 to June 2018. Data of clinicopathological characteristics, surgical and postoperative outcomes, and follow-up findings in NOSES-LAR cases were collected and retrospectively compared with those of conventional LAR to clarify the clinical benefits. The median postoperative hospital stay was lower in the double-purse NOSES group than the conventional group (6.6 vs. 7.1 days, respectively). In the conventional group, anastomotic leakage and incision site infection occurred in one patient each. In contrast, there were no complications in the double-purse group. There were no significant differences in blood loss, surgical duration, and time of the first flatus between the two groups. Additionally, “double-purse” NOSES-LAR was more economical than the conventional LAR. “Double-purse” NOSES-LAR is a safe, feasible, and minimally invasive promising procedure for LAR of RC with faster recovery, while requiring less surgical skills and lower clinical costs.

## 1. Introduction

Laparoscopic anterior resection (LAR) is widely used for rectal cancer (RC) because of the minimally invasive nature and safety of the procedure, thereby supporting its use as an alternative to open surgery [1–3]. However, current conventional laparoscopic-assisted procedures usually require additional abdominal incisions for specimen extraction and completing anastomosis, and minilaparotomy can often lead to postoperative pain, surgical site infection, incisional hernia, and poor cosmetic outcomes [4–6].

In recent years, natural orifice transluminal endoscopic surgery (NOTES) has become the focus of RC surgery. However, NOTES requires technological expertise and specialized devices, which limits its applicability in clinical practice [7, 8]. At this time, natural orifice specimen extraction surgery (NOSES) has been increasingly applied due to its advantage of a reduced risk of abdominal wounds [9–13]. There are various methods for extraction of RC specimens and digestive tract reconstruction. In combination with clinical practice, our center introduced a modified surgical procedure, called “double-purse” NOSES, for reconstruction and collection of specimens from the anus. However, the long-term efficacy of double-purse NOSES is unknown. Therefore, the aim of the present study was to evaluate the short-term efficacy of double-purse NOSES for resection of RC. This retrospective study was approved by the Ethics Committee of Huashan Hospital, Fudan University.

## 2. Materials and Methods

### 2.1. Patients

The study cohort consisted of 20 patients with RC [11 males and 9 females; median age, 63 years; age range, 40–75 years; mean body mass index (BMI), 23.5 kg/m^2^; BMI range, 19.2–27.8 kg/m^2^] who underwent complete LAR via the transanal approach in the Department of General Surgery of Huashan Hospital (affiliated to Fudan University) from October 2017 to June 2018. A diagnosis of rectal adenocarcinoma was confirmed before surgery, and all procedures were performed endoscopically. Enhanced computed tomography, magnetic resonance imaging, and other auxiliary examinations were performed to ensure no invasion of the serosal layer or distant metastases, especially to the liver or lung.

The indications for LAR with double-purse NOSES were (1) a dentate line from the lower margin of the tumor > 5 cm, (2) tumor invasion depth ≤ cT3, (3) circumferential diameter of the tumor < 5 cm, and (4) body mass index < 28 kg/m^2^. Relative contraindications were (1) tumor invasion of the serosal layer, (2) tumor diameter > 5 cm, and (3) short and thick mesentery.

According to the above indications, 20 patients who underwent traditional LAR were assigned to the control group.

Colorectal cancer in both groups was single tumor. The diameter of all patients' tumor was less than 5 cm. There were 20 cases of mass carcinoma, 15 cases of invasive carcinoma, and 5 cases of ulcerative carcinoma. There was no significant differences in age, sex ratio, BMI, tumor size, and tumor gross type between the two groups.

This retrospective study was approved by the Ethics Committee of Huashan Hospital.

### 2.2. Surgical Procedures of Double-Purse NOSES-LAR

After general surgery, the patient was placed in the lithotomy position. A curved incision was made up to the umbilicus, and a 10-mm trocar was placed. After pneumoperitoneum establishment, the abdominal cavity was explored to determine whether invasion or metastasis had occurred. Then, a 12 mm trocar was placed in the major surgical port in the right lower quadrant, and a 5 mm trocar was placed at the intersection of the level of the umbilicus and the outer edge of the right rectus abdominis. Two additional 5 mm trocars were placed symmetrically on the left side of the abdomen.

Generally, the lymphovascular trunk to the rectosigmoid colon was carefully divided and ligated. Then, the sigmoid colon and associated mesocolon were mobilized in the mediolateral direction in Toldt's fascia. Afterwards, sharp pelvic dissection with a nerve-sparing technique was performed according to the principle of total mesorectal excision. Rectal “baring” was performed at about 5 cm in the distal part of the tumor. Next, approximately 3 cm of the bowel was bared during predissection of the proximal sigmoid colon (generally 10 cm from the upper edge of the tumor). Then, the upper and lower sides of the bared area were, respectively, ligated with a blocking plier and sterilized hemp rope to prevent contamination of the surgical field, as well as tumor spread that might be caused by surgical mobilization. Afterward, an ultrasound scalpel was used to transect the bowel, and both ends were disinfected with iodine volt gauze.

**Figure 1:**
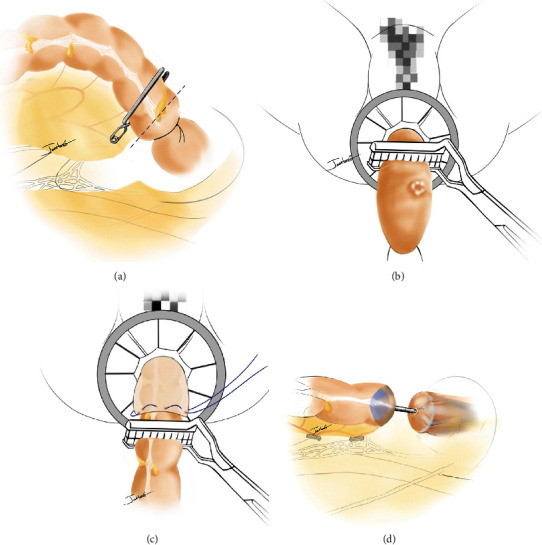


After full enlargement of the anus, the distal bowel lumen containing the tumor was pulled out through the anus using oval forceps. A large amount of iodine volt gauze was used to scrub the rectal mucosa, and then the specimen was removed with purse-string forceps after the lower edge of the incision was accurately judged. At this time, the purse-string knot (“No. 1 purse”) was not tied. Then, sponge forceps were used to pull the proximal sigmoid colon out of the pelvic cavity through the rectal stump under laparoscopic guidance. The detachable anvil of a circular stapler (CDH29; Ethicon Inc., Somerville, NJ, USA) was put into the proximal colon end, fixed firmly using the same purse-string instrument, and then returned back to the abdominal cavity. Afterward, the “No. 1 purse” of the rectal remnant was tightened moderately and knotted (note: the “No. 1 purse” should not be tightened too tightly so that the central hole can pass through the tip of the hemostatic forceps just enough). Then, this segment of the bowel lumen was reverted back into the abdominal cavity. After reestablishing the pneumoperitoneum, the circular stapler was inserted into the anus, and the central rod was penetrated from the “No. 1 purse” via the central hole with careful adjustment to finish the end-to-end anastomosis. The process is shown in Figure 1.

The pelvic cavity was flushed with a large amount of dilute iodine volts and normal saline, and then a pelvic drainage lumen was inserted through the trocar hole in the right lower abdomen to the pelvic floor. The trocar hole was subcutaneously injected with ropivacaine. The wound was cemented with biological adhesive without scar treatment.

### 2.3. Traditional Group

The procedure of the radical resection was the same as with the double-purse technique, but the reconstruction style differed. Briefly, the rectum was transected more than 2 cm distant from the lower margin of the tumor with a 60 mm endoscopic linear stapler (EC60A; Ethicon Endo-Surgery, Inc., Blue Ash, OH, USA). Subsequently, an incision about 5–6 cm was made to the lower abdomen, from which the specimen was resected and removed. After treatment with a purse-string instrument, the anvil was inserted into the proximal colonic ends. Then, the incision was sutured under laparoscopic vision to complete the end-to-end anastomosis.

### 2.4. Postoperative Treatment

Prophylactic use of antibiotics lasted in 48 h. The patient was offered water at 6–8 h after anesthesia, and a normal diet was offered after exhaust defecation was restored. Finally, the pelvic drainage tube was removed.

### 2.5. Statistical Analysis

All statistical analysis was carried out through a commercial statistical software package SPSS 22.0 by IBM. The Mann–Whitney *U* tests were used to test pairwise differences between two groups for continuous variables and for ordered categorical variables. Chi-squared tests were used for categorical variables. *P* values <0.05 were held as significant.

## 3. Results

### 3.1. Patient Characteristics and Surgical Outcomes

The baseline demographics of the two groups were comparable (Table 1). Of the 20 double-purse patients, 9 (45%) were females, and 11 (55%) were males with a median age of 63 (range, 40–75) years and mean BMI of 23.5 ± 3.0 (range, 17.9–29.8) kg/m^2^. Of the 20 patients in the conventional treatment group, 12 (60%) were males, and 8 (40%) were females with a median age of 64 (range, 38–79) years and mean BMI of 22.9 ± 2.9 (range, 16.6–30.1) kg/m^2^. No patient had critical organ dysfunction.

There was no surgery-related death, and no prophylactic enterostomy was performed intraoperatively in either group. All patients had a negative surgical margin. There were no significant differences in mean blood loss and time to first flatus. However, four patients in the conventional group complained of pain from the incision and were treated with painkillers, while none in the double-purse group has this complaint. Meanwhile, the median postoperative hospital stay was shorter for the double-purse group than the conventional group (6.7 vs. 7.8 days, respectively), although there was no statistical significance due to the small sample size. Moreover, the hospitalization costs of our new method is less than that of the traditional method. The tumor characteristics were similar between the groups (Table 1).

### 3.2. Postoperative Complications and Follow-Up

Postoperative complications are listed in Table 2. There was no instance of postoperative abdominal or anastomotic hemorrhage in either group. However, mild anastomotic leakage was observed in one patient in the traditional surgery group, which led to a pelvic infection, and one patient developed an infection of the incision site. Both complications were solved by conservative treatment. In contrast, there were no complications in the double-purse group.

All patients postoperatively diagnosed with stage III RC received adjuvant chemotherapy with capecitabine plus oxaliplatin. At a mean follow-up duration of 9.5 ± 4.2 months after surgery, all patients survived with no instance of incisional hernia at the trocar or incisional site and no case of local recurrence. However, one patient in the conventional group was diagnosed with liver metastasis at 9 months after surgery.

## 4. Discussion

With the development of the minimally invasive concept, laparoscopic-assisted radical resection has gradually replaced laparotomy as the main surgical method for the treatment of RC. However, classic laparoscopic surgery still requires an auxiliary incision into the abdomen of 4–6 cm to complete the removal of specimens, implantation of the anvil, and digestive tract reconstruction. An abdominal incision increases the risk of postoperative wound pain, resulting in delayed time to free movement and discharge, as well as incision-related complications, such as infection, intestinal adhesion, tumor implantation, etc. Thus, the advantages of laparoscopic minimally invasive surgery are obviously weakened [14–16].

Although still in the stage of clinical exploration, NOTES requires specific equipment and experience with the surgical technique. Therefore, surgery of nonincision specimen extraction for colorectal cancer can be considered as a transitional stage from traditional laparoscopic surgery to scar-free surgery. As a benefit of NOSES over the conventional method, specimens can be removed from a natural orifice, such as the vagina or rectum, without the need for an abdominal incision, resulting in a better aesthetic outcome, less postoperative pain, faster flatus, and earlier activity time, while lowering the risk of incision site infection and incisional hernia. However, specimen extraction via the vagina is only an option for female patients and may increase the incidence of postoperative complications due to incision of the posterior vaginal fornix. Thus, extraction of transrectal specimens is preferred. To date, various types of NOSES for LAR of RC have been reported [17–19]. Usually, the anvil is placed into the abdominal cavity through the anus and fixed in the proximal sigmoid ends under laparoscopic surveillance, while the distal rectum stump must be closed again for anastomosis. The authors consider that there are several deficiencies as follows: (1) the surgery is comparably complicated and requires the experience of a skilled team; otherwise, it is difficult for the surgeons to accurately determine whether the anvil is properly fixed, (2) the remnant rectal stump cannot be too short to apply the endo-GIA to close the ends, (3) there are still two weak horns around the anastomotic stoma after reconstruction, which are difficult to reinforce with sutures, and (4) increased surgical costs. The first three deficiencies are closely related to the occurrence of postoperative anastomotic leakage, which restricts the choices of the colorectal surgeons to a nonincisional technique.

To solve these problems, we introduced a modified NOSES method, called the “double-purse” procedure, which can be summarized in four steps, as follows: (1) two ligation strings (or one blocking plier) are tied to the bowel after baring around the upper surgical margin of the tumor under laparoscopic vision, and the intestine lumen is transected between the strings, which not only guarantees an adequate upper margin of the incision but also complies with the tumor-free principle. (2) The diseased bowel segment is turned over and pulled from the body through the anus. Then, the specimen is removed using purse-string forceps after the lower margin is determined under direct vision. At this point, the purse string is not tightened. (3) Oval forceps are used to drag the proximal colon out from the body via the rectal stump under laparoscopic guidance, and the anvil is planted and fixed firmly using the same “purse” method. Afterward, this proximal segment is returned to the body. (4) The purse string of the valgus rectum is tightened and knotted moderately and then reversed backed into the peritoneal cavity. Under laparoscopic guidance, the pole of the stapler is pushed out from the middle of the purse string to complete an end-to-end anastomosis. As compared with the conventional method, we consider that the “double-purse” method has the following advantages: (1) the double-purse NOSES technique requires the proximal colon to be pulled out through the stump of the rectum, so the stump should not be too long, making it more suitable for tumors in the middle or even much lower rectum. (2) The procedure of placing the anvil into the proximal colonic canal for the purse string is much easier and more reliable because it is accomplished under direct vision, as compared to the conventional method, due to defects of two-dimensional vision under laparoscopy. (3) The traditional method requires the dissociation of the rectum more distally; otherwise, the endo GIA cannot be used for exact closure of the rectal stump. However, with the “double-purse” string method, it is not necessary to dissociate too many intestinal lumens, and dispensing with the closure device can also reduce costs. (4) After reconstruction of the alimentary tract by traditional methods, an area with an insufficient blood supply (weak angle) is generally found, while the weak angle is eliminated after end-to-end anastomosis by the double-purse-string method, which might reduce the incidence of postoperative anastomotic leakage.

In this study, the short-term efficacy of total laparoscopic resection of RC with NOSES was satisfactory in all 20 cases: the median surgical duration was 120 min, the median intraoperative blood loss was 35 ml, the median time to flatus was 48 h, no patient experienced severe postoperative complications, and no tumor recurrence or metastasis was observed during the follow-up period. It is considered that several important factors are essential to obtain a satisfactory curative effect and smooth operation in addition to the above procedure, which include (1) skill with the laparoscopic technique for LAR during NOSES to determine the length of the specimen, baring of the intestinal lumen, and completion of the anastomosis, which requires overcoming the anatomic dislocation of laparoscopic vision, and tacit cooperation between the operator and camera man. (2) Selection of appropriate patients (i.e., tumor distance from the dentate by 5–15 cm, no invasion of the serosa or neighboring structures (cT1–T3), tumor diameter < 5 cm, BMI < 28 kg/m^2^, sufficient length of the sigmoid colon, and capacity to tolerate laparoscopic surgery). Patients who conform to the foregoing criteria can be considered candidates. (3) The purse string of the distal rectum stump must be tightened properly to facilitate puncture of the central rod of the circular staple. (4) Good bowel preparation and intraoperative sterile, tumor-free operations, such as ligation of the bowl before transection, iodine-volt cleaning of the rectum, and saline flushing of the pelvic cavity.

## 5. Conclusions

In conclusion, based on the principles of sterility, tumor-free surgery, and radical cure, the proposed double-purse NOSES technique for RC is more economical and might reduce the risk of postoperative complications related to the incision in some select patients. Meanwhile, the technique is safe and feasible with satisfactory short-term efficacy and is suitable for widespread application in a number of colorectal cancer treatment centers. Finally, the indications for this approach should be met, while prospective randomized controlled clinical studies are warranted to evaluate the long-term benefits.

## Figures and Tables

**Table 1:** Patient demographics, surgical outcomes, and tumor characteristics of both groups.

Characteristic	Double-purse NOSES	Conventional	*P*
Patient demographics			
Age, years (range)	63 (40-75)	64 (38-79)	0.547
Sex ratio, male/female	11/9	12/8	0.818
Body mass index, BMI (kg/m^2^)	23.5 ± 3.0	22.9 ± 2.9	0.859
Tumor size	2.5 ± 0.9	2.3 ± 0.7	0.647
Tumor number	Single	Single	
Tumor gross type			
Mass carcinoma	10	10	
Invasive carcinoma	8	7	
Ulcerative carcinoma	2	3	
Surgical outcomes			
Surgical duration, min	129.7 ± 27.5	132.4 ± 25.8	0.788
Blood loss, mL	56.5 ± 24.6	54.2 ± 21.5	0.474
Time to first flatus, d	2.4 ± 0.8	2.7 ± 1.3	0.252
Postoperative hospital stay, d	6.9 ± 0.6	7.8 ± 3.2	0.158
Hospitalization costs^∗^	4.3 ± 0.5	5.3 ± 0.5	<0.05
Retrieved LNs, *n*	20.7 ± 5.9	22.4 ± 6.0	0.550
Pathologic findings (*n*) b			
T1/T2/T3/T4	6/8/6/0	5/8/5/2	0.680
N0/N1/N2/N3	14/5/1/0	12/7/1/0	0.645
TNM stage I/II/III/IV	8/6/6/0	5/7/8/0	0.664

^∗^10 thousand yuan.

**Table 2:** Postoperative complications.

Characteristic	Double-purse NOSES	Conventional
Intraperitoneal or digestive tract hemorrhage, *n*	0	0
Intraabdominal infection or abscess, *n*	0	1
Incision site infection, *n*	0	1
Anastomotic leakage, *n*	0	1
Anastomotic stenosis, *n*	0	0

## Data Availability

The data used to support the findings of this study are available from the corresponding author upon request.
